# Trumped Treatment?: BPA Blocks Effects of Breast Cancer Chemotherapy Drugs

**Published:** 2009-02

**Authors:** Julia R. Barrett

Widespread human exposure to the chemical bisphenol A (BPA) has resulted from its use in a diverse array of consumer products. Research on the potential health effects of BPA has focused on the chemical’s ability to mimic or block natural estrogen. In animal studies, prenatal exposure to BPA increased susceptibility to mammary cancer in adulthood. However, studies of adult animals and cell cultures have had mixed results, and even less certain is how BPA might influence established breast cancer and its treatment. A new cell culture study is the first to show that BPA, at concentrations comparable to those found in the general population, reduces the efficacy of chemotherapy drugs in breast cancer cells, apparently by altering expression of proteins involved in apoptosis, or programmed cell death **[*EHP*** **117:175–180; LaPensee et al.]**.

The study used estrogen-responsive T47D cells and estrogen-insensitive MDA-MB-468 cells. Cells were pretreated with BPA and then incubated alone or with one of several concentrations of added doxorubicin, cisplatin, or vinblastine, commonly used chemotherapy drugs. BPA concentrations (1 and 10 nM) were comparable to levels documented in human milk and blood (0.5–40 nM). In some experiments, BPA pretreatment was preceded by addition of either ICI or PHTPP, compounds that block classical estrogen receptor-α and -β (ERα and ERβ, respectively). In addition to assessing cell survival, the researchers determined genetic expression and concentrations of the antiapoptotic proteins Bcl-2, Bcl-xL, and survivin, as well as classical and nonclassical estrogen receptors.

The researchers found in T47D cells that BPA partially or completely blocked the effects of all doses of doxorubicin and cisplatin but only the lowest dose of vinblastine, with similar results in MDA-MB-468 cells. BPA by itself bolstered cell viability (i.e., chemoresistance) in T47D cells but not in MDA-MB-468 cells. Neither ICI nor PHTPP affected the ability of BPA to block doxorubicin’s actions in T47D cells, and BPA was able to mediate the drugs’ effects in MDA-MB-468 cells. These observations together suggest that BPA does not act via ERα or ERβ, helping to account for its effects on ER-insensitive cells.

Given previous research that demonstrates a high binding affinity of estrogen-related receptor-γ (ERRγ) to BPA **[*EHP*** **116:32–38 (2008)]**, the researchers speculate that ERRγ, expressed by both cell lines in their study, is the most likely target for BPA binding. Based on their finding that BPA combined with doxorubicin increased levels of Bcl-2 and Bcl-xL, the researchers conclude that derailment of apoptosis could be a mechanism—perhaps common to all three drugs—by which BPA may inhibit the efficacy of breast cancer drugs. Both BPA binding to ERRγ and the compound’s role in apoptosis should be further examined in future research.

## Figures and Tables

**Figure:**
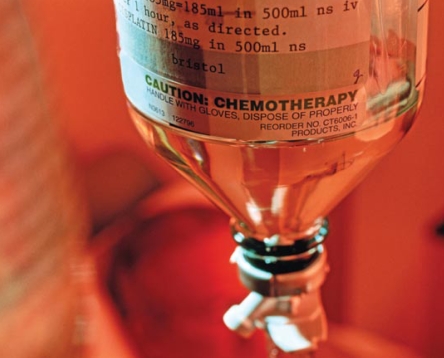
Bisphenol A prevented chemotherapy drugs from inducing apoptosis in cultured breast cancer cells.

